# Construction of Large Cranial Windows With Nanosheet and Light-Curable Resin for Long-term Two-Photon Imaging in Mice

**DOI:** 10.21769/BioProtoc.5373

**Published:** 2025-07-05

**Authors:** Taiga Takahashi, Yu Makino, Yosuke Okamura, Tomomi Nemoto

**Affiliations:** 1 Graduate School of Advanced Engineering, Tokyo University of Science, Niijuku, Katsushika, Tokyo, Japan; 2 Biophotonics Research Group, Exploratory Research Center on Life and Living Systems (ExCELLS), National Institutes of Natural Sciences, Higashiyama, Myodaiji, Okazaki, Aichi, Japan; 3 Division of Biophotonics, National Institute for Physiological Sciences, National Institutes of Natural Sciences, Higashiyama, Myodaiji, Okazaki, Aichi, Japan; 4 Micro/Nano Technology Center, Tokai University, Kitakaname, Hiratsuka, Kanagawa, Japan; 5 Department of Applied Chemistry, School of Engineering, Tokai University, Kitakaname, Hiratsuka, Kanagawa, Japan; 6 Course of Applied Science, Graduate School of Engineering, Tokai University, Kitakaname, Hiratsuka, Kanagawa, Japan

**Keywords:** Cranial window, Two-photon imaging, Mouse, Nanomaterial, Neuroscience, Surgery, Fluoropolymer, Chronic imaging

## Abstract

In vivo two-photon imaging of the mouse brain is essential for understanding brain function in relation to neural structure; however, its application is limited by the size and mechanical stability of conventional cranial windows. Here, we present the procedure of a large-scale cranial window technique based on the nanosheet incorporated into light-curable resin (NIRE) method. This approach utilizes a biocompatible polyethylene-oxide-coated CYTOP (PEO-CYTOP) nanosheet combined with light-curable resin, allowing the window to conform to the brain’s curved surface. The protocol enables long-term, high-resolution, and multiscale imaging—from subcellular structures to large neuronal populations—in awake mice over several months.

Key features

• This protocol establishes large-scale cranial windows by combining a flexible, biocompatible PEO-CYTOP nanosheet with light-curable resin.

• The large-scale cranial window provides long-term optical transparency and mechanical stability, enabling chronic in vivo imaging with minimal motion artifacts.

• This approach facilitates multiscale two-photon imaging—from subcellular structures to large-scale neural networks—in awake mice.

## Background

Understanding complex brain functions such as neuroplasticity, learning, and disease progression requires long-term, high-resolution in vivo imaging of the mouse brain. Conventional cranial window techniques employing glass coverslips are often limited by small imaging fields [1,2]. To overcome this limitation, cranial windows made of curved transparent materials [3–5] have been developed to enable large-scale imaging. However, these windows rely on pre-molded materials and their shape is fixed, posing a risk of compressing the brain surface. Previously, we proposed the large-scale cranial window using a PEO-CYTOP nanosheet [6,7], which offers several advantageous properties, including strong adhesion, flexibility, and optical transparency [8–10]. This cranial window using PEO-CYTOP nanosheet enables in vivo two-photon imaging with a broad field of view and high spatial resolution. However, it does not adequately suppress motion artifacts in awake animals and remains too fragile for long-term imaging due to intracranial pressure fluctuations and mechanical stress from animal behavior.

To address these challenges, we developed the nanosheet incorporated into light-curable resin (NIRE) method [11], which combines a flexible, biocompatible PEO-CYTOP nanosheet with a transparent, light-curable resin to seal and protect the brain surface. This combination not only conforms to the brain’s curved surface but also provides mechanical stability through the resin and reduces inflammatory responses via the nanosheet. As a result, this protocol enables multiscale imaging and offers a robust tool for longitudinal studies of neural network dynamics.

## Materials and reagents

**Reagents**

1. Perfluoro (1-butenyl vinyl ether) polymer (CYTOP) (AGC, catalog number: CTX-809SP)

2. Perfluorotributylamine (AGC, catalog number: CT-Solv.180)

3. PVA (MW, 22,000) (MP Biomedicals, catalog number: 9002-89-5)

4. Sylgard 184 Silicone Elastomer kit (Dow Chemical, catalog number: 4019862)

5. Hexane (Kanto Chemical, catalog number: 18041)

6. Toluene (Kanto Chemical, catalog number: 40180)

7. 2-(Methoxy(polyethyleneoxy)propyl) trichlorosilane (MW, ~538) (Gelest, catalog number: SIM6492.66)

8. GLYCEOL injection (TAIYO Pharma, catalog number: 2190501A5064)

9. Xylocaine 2% (Dentsply Sirona, catalog number: 66312-176-16)

10. Isoflurane (FUJIFILM Wako Pure Chemical Corporation, catalog number: 26675-46-7)

11. Norland optical adhesive NOA 83H (Norland Products Inc., catalog number: 55-583)

12. Ionosit baseliner (DMG, catalog number: 5801-724007)

13. Dental adhesive resin cement super-bond C&B bulk-mix (Sun Medical, catalog number: 204610557)

14. Unifast II (GC International AG, catalog number: 8200960)

15. Tarivid ophthalmic ointment 0.3% (Santen Pharmaceutical, catalog number: 084130211)

16. Aron Alpha gel (TOAGOSEI, catalog number: GEL-EX-20)

17. Spongel (LTL Pharma, catalog number: 919100716)

18. Utility wax (GC, white, catalog number: 70896000)

**Laboratory supplies**

1. Glass dropper (AS ONE, catalog number: 2-2045-01)

2. Diamond cutter (AS ONE, catalog number: 6-539-01)

3. Teflon Petri dish (AS ONE, catalog number: NR0213-004)

4. Nonwoven fabric substrate (disposable tea filter bag made from polyethylene and polypropylene) (DAISO, catalog number: H-070 No.475)

5. Drill bit #140 Φ1.4 (Minitor, catalog number: AD1407)

6. Scalpel (FEATHER Safety Razor, No.10, catalog number: 8-8386-01)

7. Scissors (Fine Science Tools, catalog number: 14090-09)

8. Tweezers (Fine Science Tools, catalog number: 11251-20)

9. Aluminum plate (NANYO SHOKAI, catalog number: SUS304)

10. Disposable heating pad mini (Lotte, catalog number: 104602)

11. Mixing paper (CG, No.1, catalog number: 8203389)

## Equipment

1. Silicon wafer (200 nm SiO_2_ coated) (KST World)

2. Spin coater (Mikasa, model: MS-A100)

3. Sterilizer (NAVIS, model: FV-209B)

4. Plasma cleaner (Harrick Plasma, model: PDC-32G)

5. Thermal oven (AS ONE, model: AVO-250NB)

6. Stylus profilometer (Bruker, model: DektakXT)

7. Contact angle meter (Kyowa Interface Science, model: DMe-211)

8. Head holder for mice (NARISHIGE, model: SG-4N)

9. Ultimate XL (NAKANISHI, model: Y141446)

10. Anesthetic vaporizer (Muromachi Kikai, model: MK-A110D)

11. Suction pump (Markos Mefar, model: SP30)

12. UV-LED (OptoSupply, model: OSV1XME3E1S)

13. LED driver (OptoSupply, model: OSMR16-W1213)

14. LED control board (Bit Trade One, model: ADULEDB)

15. Board case (TAKACHI electronics enclosure, model: PF10-4-10W)

16. Glass diffuser (Thorlabs, Ø1" unmounted N-BK7 ground 1500 grit, model: DG10-1500)

## Procedure

**A. Prepare solutions for the fabrication of the PEO-CYTOP nanosheet**

1. Dissolve CYTOP in perfluorotributylamine to a final concentration of 30 mg/mL.

*Note: The required volume depends on the sheet size; 0.5 mL is sufficient for a 40 × 40 mm^2^ nanosheet.*

2. Dissolve PVA (MW, 22,000) in distilled water at 10 mg/mL. Use a volume equivalent to that of CYTOP solution.

3. Mix Sylgard 184 elastomer base and curing agent in a 10:1 weight ratio for a few minutes at room temperature. Dilute to 1.25 wt% in hexane, and store in a sealed glass vial.

**CAUTION**: Hexane is highly flammable and neurotoxic. Use in a fume hood with nitrile gloves; store in an explosion-proof refrigerator.

*Note: To prevent solvent evaporation, store at 4 °C and use within one week.*

**B. Fabricate PEO-CYTOP nanosheet**

1. Using a glass dropper, apply the PVA solution onto the silicon wafer, then spin-coat it at 4,000 rpm for 20 s to form a sacrificial layer. The term "sacrificial layer" refers to a layer that can be dissolved in water during the freestanding process, allowing the PEO-CYTOP nanosheet to detach from the silicon wafer.

*Notes:*

*1. The spin-coating substrate comprises a silicon wafer with a 200 nm silicon oxide layer. The crystal structure of the silicon wafer is not critical. The wafer, originally four inches in diameter, is manually cut into appropriately sized squares using a diamond cutter and ruler, depending on the required dimensions of the nanosheet and the cranial window. For improved accuracy, a precision diamond wafer scriber is recommended when available.*

*2. The specific volume of solution applied to the wafer is not critical, as it does not influence the final coating thickness. However, for a uniform coating, the solution should fully cover the wafer surface before spin-coating. Typically, 0.5 mL of solution is sufficient to coat a 40 × 40 mm^2^ silicon wafer. This guideline also applies to steps B5 and B6 regarding solution volume.*

*3. A minimum waiting period of approximately 2–3 min between each solution application is recommended. Typically, solvents used in each step (PVA:water; CYTOP solvent; silicone/polydimethylsiloxane (PDMS) solvent:hexane) mostly evaporate during spin-coating. This brief interval ensures that solvents have sufficiently evaporated, preventing any unwanted interactions between layers and ensuring reproducibility of the coating quality.*

2. Dispense the CYTOP solution onto the PVA-coated substrate and spin-coat at 4,000 rpm for 60 s.

3. Apply the silicone solution onto the CYTOP-coated substrate and spin-coat at 4,000 rpm for 20 s at room temperature.

4. Cure the multilayer composite at 80 °C for 2 h.

5. Subject the composite to oxygen plasma treatment at 11 W (medium power setting) for 60 s to enhance surface hydrophilicity.

*Note: Oxygen plasma consists of a combination of oxygen ions, radicals, ozone, and neutral oxygen atoms, which facilitates the introduction of hydroxyl groups onto PDMS, rendering the surface hydrophilic.*

6. Prepare a 2 mM solution of PEO-silane [2-(methoxy(polyethyleneoxy)propyl) trichlorosilane] in toluene within a Teflon Petri dish (ϕ: 150 mm, height: 30 mm, capacity: 300 mL).

7. Submerge the substrate in the PEO-silane solution within the Teflon Petri dish for 1 h to achieve long-term stabilization of the hydrophilic surface.

*Note: Because PEO-silane is highly susceptible to hydrolysis, it should be prepared immediately before use. While the precise volume of the PEO-silane solution is not critical, the substrate must be fully immersed. For the specified Teflon Petri dish, 100 mL of solution is sufficient for processing four 40 × 40 mm^2^ nanosheets.*

8. Immerse the substrate in water to dissolve the sacrificial layer, allowing the PEO-CYTOP nanosheet to detach and float on the surface.

9. Transfer the floating PEO-CYTOP nanosheet from the water surface onto a nonwoven fabric using tweezers, ensuring that the hydrophilic side faces outward.

*Notes:*

*1. The nonwoven fabric was sterilized using an 8 W UV sterilizer for 30 min prior to use.*

*2. Applying water droplets can distinguish the hydrophilic and hydrophobic surfaces. If the droplet forms a spherical bead, the surface is hydrophobic; if it spreads evenly, the surface is hydrophilic.*

10. Allow the PEO-CYTOP nanosheet to dry on the nonwoven fabric overnight (minimum 12 h).

*Notes:*

*1. PEO-CYTOP nanosheets should be stored in a dry, dust-free container at room temperature.*

*2. This protocol typically yields PEO-CYTOP nanosheets with a thickness of approximately 130nm. If the nanosheet is significantly thicker (e.g., ~300nm), structural coloration may become visible due to optical interference, making such deviations detectable by eye. For precise evaluation, we recommend using a stylus profilometer (e.g., DektakXT, Bruker) to measure nanosheet thickness. Surface hydrophilicity can also be assessed using a contact angle meter (e.g., DMe-211, Kyowa Interface Science) following plasma and silane treatment.*

**C. Prepare equipment for making the large-scale cranial window**

**Figure 1.**
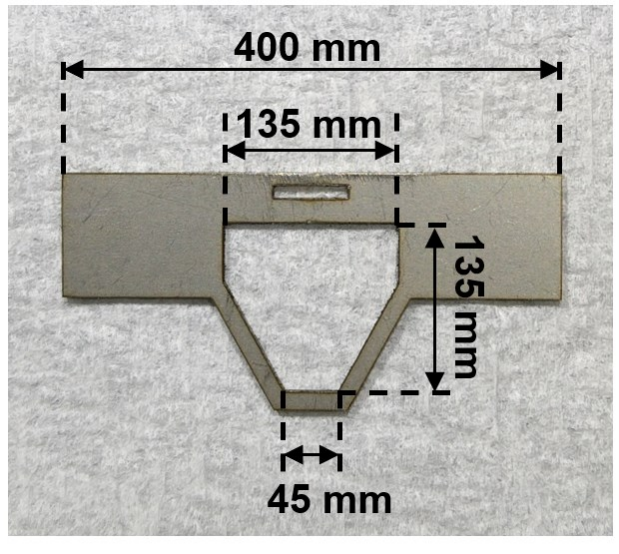
Head plate to immobilize the mouse’s head

**Figure 2.**
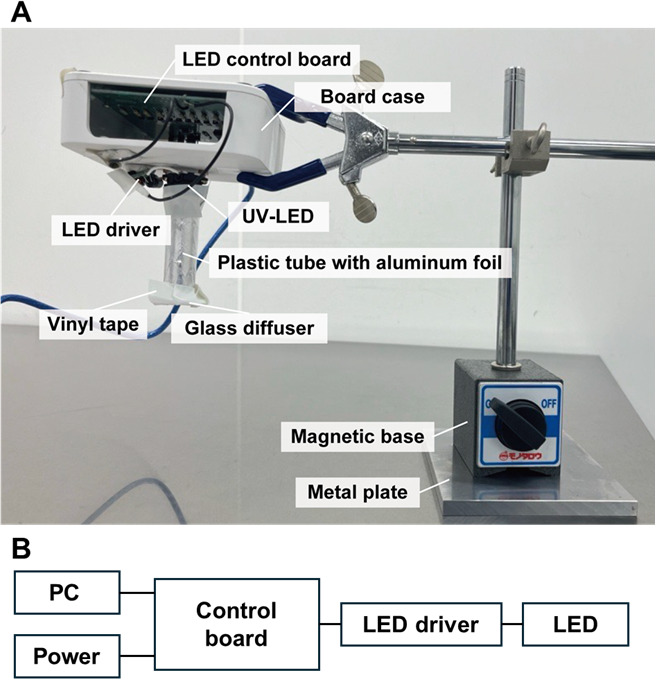
Custom-built UV-LED irradiation system for curing light-curable resin in the nanosheet incorporated into light-curable resin (NIRE) method. (A) The irradiation system consists of a UV-LED light source mounted in a custom housing. The LED control board is enclosed within a board case, and the UV-LED is connected to the control board via an LED driver. The light is directed downward through a plastic tube wrapped with aluminum foil to minimize light leakage. A glass diffuser is attached to the tip of the tube to ensure even light distribution. The entire setup is mounted on a metal arm with a magnetic base. (B) Wiring diagram of the UV-LED irradiation system.

1. Fabricate the head plate from a 1-mm-thick aluminum plate using machine tools or a machine shop (Figure 1).

2. Construct a programmable UV irradiator consisting of an LED control board, a UV-LED, a board case, and a glass diffuser (Figure 2).

3. Sterilize all surgical instruments and equipment, including the scalpel, scissors, tweezers, drill bit, head plate, and head holder, using 70% ethanol.

4. Prepare GLYCEOL injection (anti-edema agent) (15 μL/g body weight) and 200 μL of xylocaine (local anesthetic), each in separate syringes.

**D. Remove the skull of the mouse**

**CRITICAL**: All animal procedures must be conducted in accordance with the ethical guidelines of your institution. The selection and administration of anesthetics and other drugs should be adjusted as necessary for each step, following the approved protocols.

1. Anesthetize an adult mouse (≥8 weeks old) with isoflurane using an inhalation anesthetic system.

*Note: For anesthesia induction, administer isoflurane at 3%. After induction, maintain anesthesia at 1.5% isoflurane throughout the procedure, including the imaging session.*

2. Disinfect the scalp hair using cotton swabs soaked in 70% ethanol.

3. Apply ophthalmic ointment to the surface of the eye to prevent corneal desiccation.

4. Remove the scalp hair.

a. Carefully shave the hair with a razor blade.

b. Wipe the remaining hair with 70% ethanol-soaked cotton swabs.

**Figure 3.**
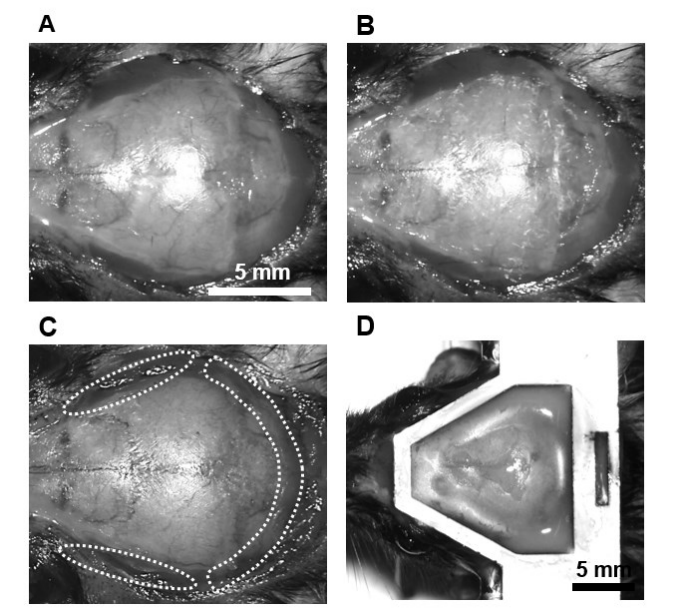
Procedure for the attachment of the head plate. (A) Incise the skin over the skull using surgical scissors after removing the scalp hair. (B) Remove the periosteum from the skull surface. (C) Separate the temporal and occipital muscles from the skull. (D) Attach the head plate to the mouse skull.

5. Secure the mouse's head in a stereotaxic frame.

6. Administer a few drops of xylocaine as a local anesthetic onto the skin.

7. Allow 3 min for the local anesthesia to take effect.

8. Incise the skin over the dorsal skull using surgical scissors, exposing the parietal, frontal, temporal, and occipital regions.

9. Apply additional xylocaine drops onto the exposed skull.

10. Wait for 3 min for the anesthetic to take effect.

11. Carefully remove the periosteum from the skull surface using a scalpel or forceps (Figure 3B, Video 1).

**CRITICAL**: Ensure complete removal of the periosteum. For a large cranial window, secure strong fixation of the head plate.

12. Separate the temporal and occipital muscles from the skull using fine forceps (Figure 3C, Video 1).

**Video 1.**
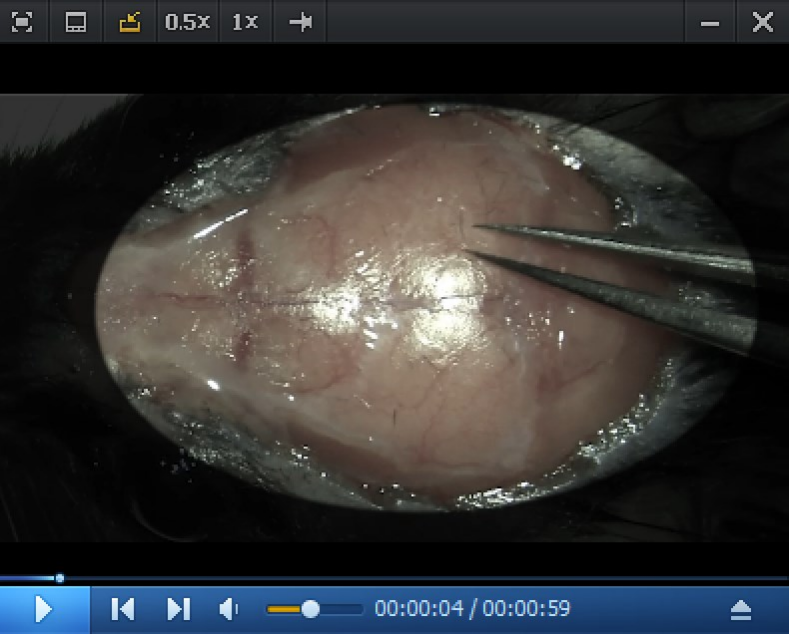
Remove the periosteum and separate the temporal and occipital muscles from the skull

13. Fix the head plate (Figure 3D).

a. Align the head plate parallel to the transverse plane of the skull.

b. Secure the head plate using dental resin.

c. Allow the dental resin to cure for 15 min.

**CRITICAL:** Confirm that the head plate is firmly attached to prevent detachment during imaging.

14. Administer GLYCEOL injection (15 μL/g body weight) intraperitoneally to reduce intracranial pressure.

**CRITICAL:** Lowering intracranial pressure facilitates skull removal by increasing the space between the brain tissue and the skull.

15. Skull thinning and grooving (Video 2):

a. Drill the skull to create a groove around the intended craniotomy area.

b. Gradually thin the bone until it can be easily depressed using light pressure with tweezers.

**CRITICAL:** Avoid applying vertical pressure with the drill bit, as this may damage brain tissue. Instead, move the drill horizontally along the groove in a circular motion.

*Notes:*

*1. The cranial window size can be adjusted as needed. Unlike glass coverslips, PEO-CYTOP nanosheets can be trimmed to any desired shape.*

*2. The area just above the bregma and lambda should be especially thin because it has strong connections between the skull and the sagittal sinus.*

**Figure 4.**
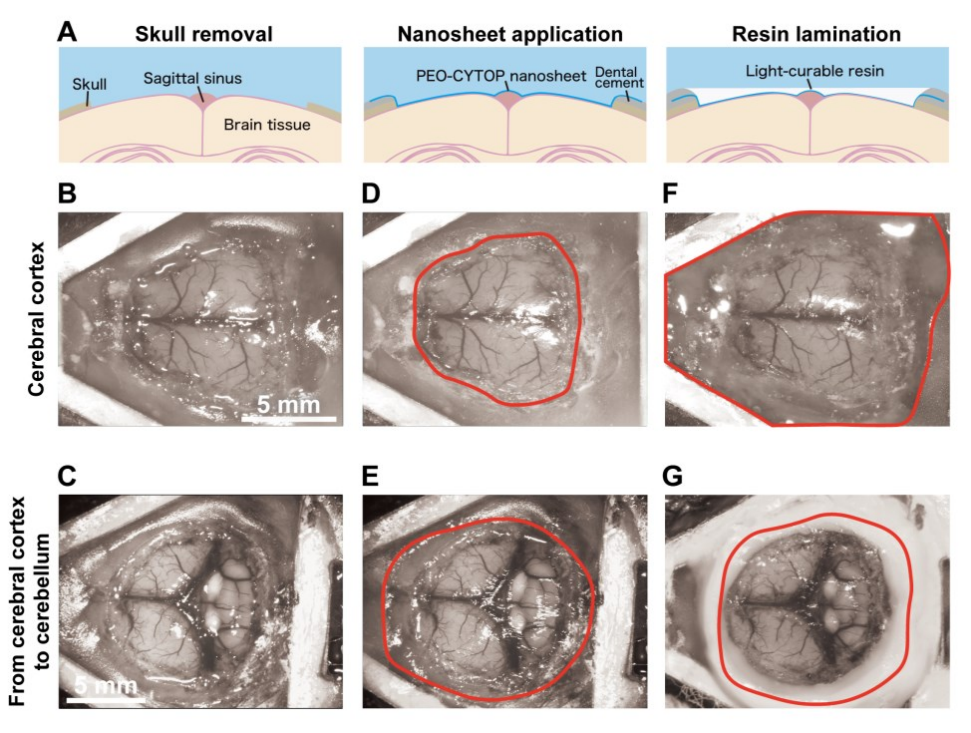
Step-by-step procedure for constructing a large-scale cranial window using the nanosheet incorporated into light-curable resin (NIRE) method. (A) Schematic illustrations of the cranial window construction procedure using the NIRE method. (B, C) Exposed brain surface after skull removal, ranging from the cerebral cortex (B) to the cerebellum (C). (D, E) Application of the PEO-CYTOP nanosheet to the exposed brain surface. The nanosheet closely conforms to the curved brain surface, covering both cortical (D) and cerebellar (E) regions. The red solid line indicates the edge of the nanosheet. (F, G) Lamination of light-curable resin to seal the nanosheet and stabilize the cranial window. The red solid line indicates the edge of the light-curable resin. Scale bars, 5 mm.

16. Skull removal (Figure 4B,C, Video 2):

a. Soak the drilled groove with saline solution.

b. Insert tweezers between the skull and brain surface, then gently lift the skull.

**CRITICAL**: Maintain tweezers parallel to the brain surface to prevent damage to the brain tissue. Slowly lift the bone while soaking it in saline to prevent tearing blood vessels attached to the skull, which could cause severe sagittal sinus injury.

**Video 2.**
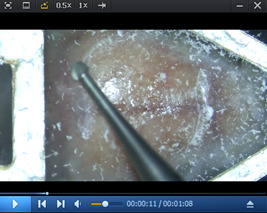
Remove the skull and adjust the edge of the PEO-CYTOP nanosheet

17. To stop the bleeding, use forceps to hold a saline-soaked Spongel and gently press it against the bleeding site to promote hemostasis.

**CRITICAL**: Minor bleeding from the sagittal sinus is expected, as the overlying skull must be removed. However, Spongel can effectively stop bleeding unless major blood vessels are severely damaged.

**E. Sealing brain tissue with PEO-CYTOP nanosheet (Figure 4D, E)**

1. Trim the PEO-CYTOP nanosheet and nonwoven fabric using scissors to cover the exposed brain region.

*Note: A slightly oversized nanosheet is held above the exposed brain region—without touching the tissue—to visually estimate the appropriate size. It is then carefully trimmed to fit the target area.*

2. Apply a few drops of saline to the brain surface, then remove excess fluid using a paper towel or suction pump.

3. Position the PEO-CYTOP nanosheet with the hydrophilic side facing the brain to ensure strong adhesion and minimize bleeding.

4. Gently press the nanosheet onto the brain surface using tweezers over the nonwoven fabric.

**CRITICAL:** Avoid excessive pressure to prevent damage to and bleeding from brain tissue.

5. Once attached, carefully peel off the nonwoven fabric using tweezers.

6. Trim the edge of the PEO-CYTOP nanosheet with tweezers (Video 2).

**CRITICAL**: Ensure the nanosheet edge remains a few millimeters away from the exposed brain area to provide sufficient space for adhesion.

7. Secure the nanosheet edges to the skull surface coated with glue (Aron Alpha Gel).

**CRITICAL**: If the brain surface swells due to elevated intracranial pressure, reduce the pressure by administering GLYCEOL injection (5 μL/g body weight) intraperitoneally before fixation.

8. Apply dental resin (Unifast II or Ionosit baseliner) over the glue layer to create a barrier for retaining light-curable resin before curing.

**F. Lamination of PEO-CYTOP nanosheets with light-curable resin (Figure 4F, G)**

1. Apply light-curable resin onto the nanosheet.

*Note: To avoid leaving air bubbles in the observation window, first discard a few drops of the light-curing resin in another place, then drop it on the nanosheet.*

2. Irradiate the resin with UV light under optimal conditions.

*Notes:*

*1. In NOA83H, we use the following pattern: 365 nm excitation light (10 mW) for 2 s every 30 s for 3 min, followed by UV irradiation for 10 s every 30 s for 7 min. The pattern should be changed depending on the types of light-curable resin and UV irradiator.*

*2. Critical parameters for proper curing with the UV irradiation system are the irradiation time and power. Although continuous exposure provides efficient curing, it also causes a significant increase in temperature, which may damage brain tissue. To avoid this, it is essential to use intermittent irradiation with intervals ranging from a few to several tens of seconds.*

**Figure 5.**
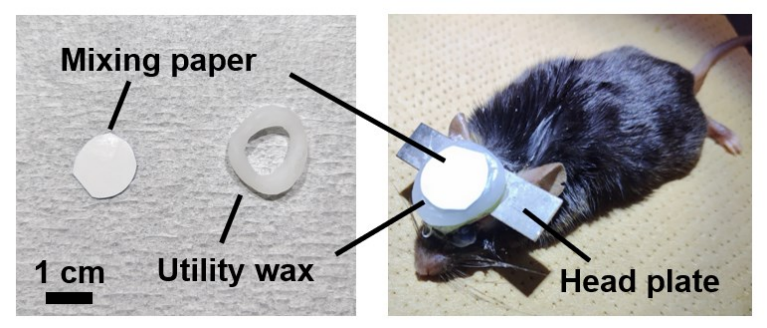
Cranial window protected using mixing paper and utility wax

3. Place utility wax around the edge of the cranial window, put a mixing paper on the wax over the cranial window, and secure it with vinyl tape to maintain the transparency of the cranial window long-term and prevent damage in a cage (Figure 5).

**CRITICAL**: If debris adheres to the surface, remove it using an air duster or by dropping water and using a suction pump. If it remains, gently wipe the surface with a cotton swab while moistening the surface with water. This procedure should be done the day following the experiment because, as curing progresses, the surface becomes less prone to damage.

**CRITICAL**: Avoid contact between the wax or paper and the surface of the cranial window to prevent deterioration of its transparency.

*Note: The mixing paper—a silicone-coated, water-resistant sheet typically used for mixing dental resin—is placed together with dental wax between the cranial window and vinyl tape to prevent adhesion during long-term protection. Other types of thick, non-stick paper with similar properties may also be used as substitutes.*

## Validation of protocol

This protocol was validated in the following research article:

- Takahashi et al. [11]. Large-scale cranial window for in vivo mouse brain imaging utilizing fluoropolymer nanosheet and light-curable resin. Commun Biol.

**Figure 6.**
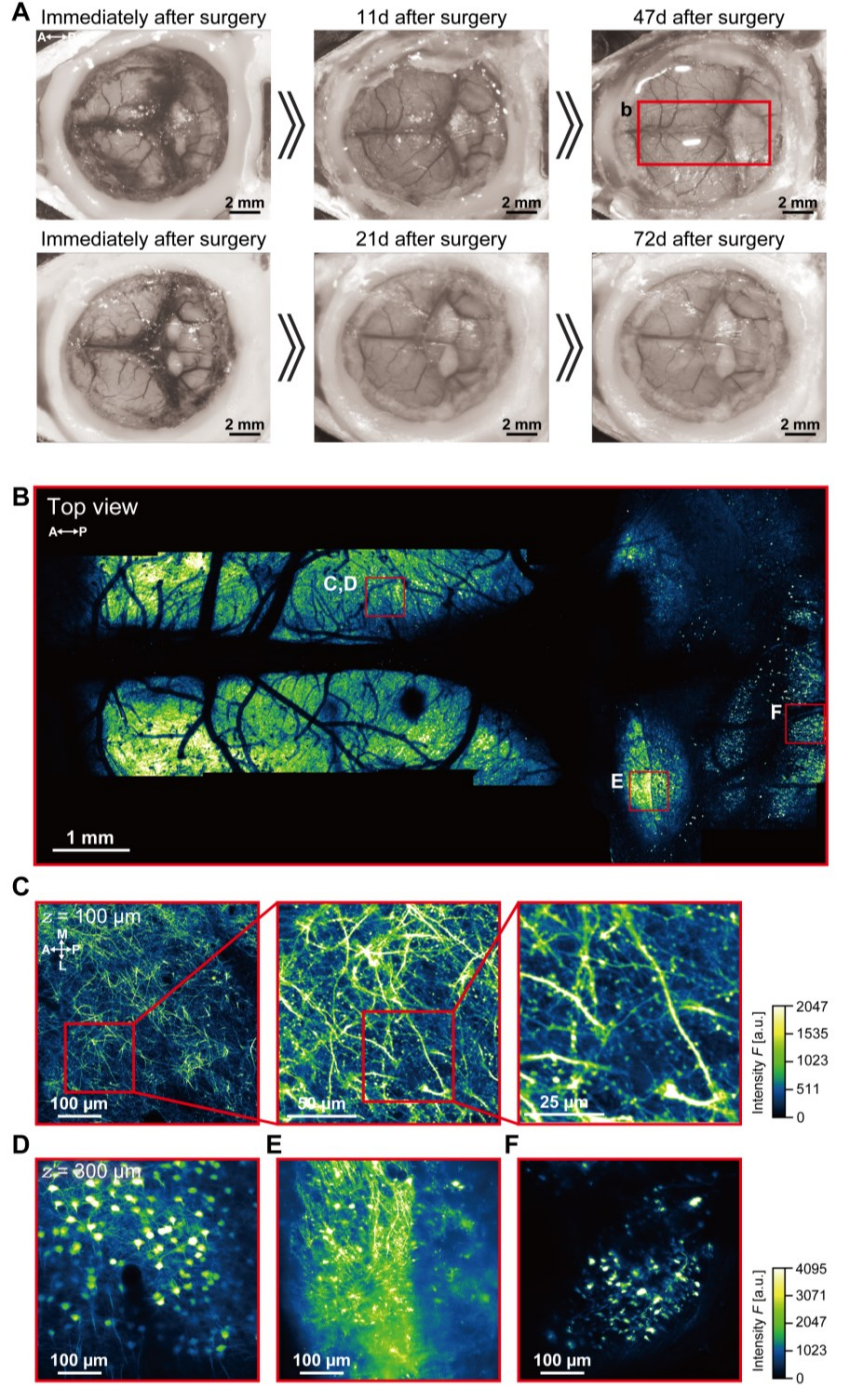
Large-scale imaging of the cerebral cortex and cerebellum using the nanosheet incorporated into light-curable resin (NIRE) method. (A) Time-lapse photomicrographs of a large cranial window covering the cerebral cortex and cerebellum of Thy1-YFP-H mice [12]. The red solid box indicates the region of panel B. (B) Large field-of-view tiled image derived from the maximum intensity projections of 12 three-dimensional stacks acquired through the cranial window in panel A. The red boxes demarcate the regions shown in panels C–F. (C) Cross-sectional XY image of axons, apical dendrites, and spines 100 µm below the cortical surface within the red box in panel B. (D) Cross-sectional XY image of a soma 450 µm below the cortical surface within the red box in panel B. (E) Cross-sectional XY image of axons, dendrites, and somata in the inferior colliculus within the red box in panel B. (F) Cross-sectional XY image of cerebellar granular cells within the red box in panel B. Olympus XLFLUOR4X/340 × 4/0.28 NA air-immersion objective lens was used in panel B. Nikon Apo LWD × 25/1.10 NA water-immersion objective lens was used in panels C–F. The directions are indicated as A (anterior), P (posterior), M (medial), and L (lateral).

The process of nanosheet fabrication, craniotomy, and sealing of the nanosheet was described in Takahashi et al. [6,7] (see Figure 4B, C). The lamination process using light-curable resin in the NIRE method was described by Takahashi et al. [11] (see Figure 1 and the “Lamination of PEO-CYTOP nanosheets with light-curable resin” sections in the Methods section of the cited article). Long-term transparency of cranial windows using the NIRE method was evaluated in Takahashi et al. [11] (Figure 6A). In addition, we compared the degree of motion artifacts under anesthetized vs. awake conditions for different cranial window materials, confirming that the NIRE method can suppress motion artifacts (see Supplementary Figure 6 and the “Analysis of FOV displacement” section of the Methods section in [11]). Large-scale in vivo two-photon imaging was performed covering from the cerebral cortex to the cerebellum (Figure 6), demonstrating the window’s stability and transparency (see also Figures 2 and 4–6 and Supplementary Figures 8 and 9 in [11]).

## General notes and troubleshooting

**General notes**

1. Disinfect instruments and the workbench with 70% ethanol to maintain long-term transparency of cranial windows. Sterilize nanosheets using UV light or ethylene oxide gas (EOG).

2. When drilling the skull, use a suction device to remove bone dust frequently. In addition, avoid drilling continuously as it may heat up the drilled area. Periodically apply saline to the drilling site to cool it and prevent tissue damage.

3. To prevent the brain tissue from drying out, apply saline onto the exposed brain surface.

4. Use isoflurane at 3% during induction and 1.5% for maintenance. Constructing the cranial window requires several hours. During this period, the mouse remains under anesthesia, which results in a reduction in body temperature. To mitigate this, a disposable heating pad should be placed under the mouse’s abdomen to maintain body temperature throughout this period.

5. When using the drill and forceps, always handle them parallel to the brain surface. Using them vertically increases the risk of damaging brain tissue.

**Troubleshooting**

Problem 1: The skull is difficult to remove.

Possible cause: Insufficient drilling. Connection between the skull and the sagittal sinus remains.

Solution: Adequately thin the skull to <20µm [13], particularly at the corners of the cranial window and in the regions above bregma and lambda. In the best cases, a small amount of cerebrospinal fluid may seep out through microcracks of the drilled area, without any bleeding.

Problem 2: Bleeding from the sagittal sinus occurred.

Possible cause: The sagittal sinus was damaged when removing the skull.

Solution: As noted in Problem 1, bleeding from the sagittal sinus may occur even with proper procedure. In such cases, place a piece of Spongel soaked in saline gently on the bleeding site. By indirectly suctioning the blood-mixed saline using an aspirator, hemostasis can be achieved. Before removing the Spongel, refill the brain surface with saline.

Problem 3: The cranial window gets increasingly cloudy as time passes.

Possible cause: Connective tissue and dura mater thickening occurred primarily around the site of brain tissue injury. Postoperative bleeding occurred extensively.

Solution: When handling the nanosheet, hold it by the edge. It is also important to perform proper hemostasis during the surgery. It is recommended to perform the first imaging within 2 weeks post-surgery; if no cloudiness is observed at that time, it is generally expected that the window will remain clear for several more months.

Problem 4: The light-curable resin does not harden sufficiently.

Possible cause: Incomplete UV exposure/curing.

Solution: Reapply UV irradiation. However, excessive repetition may cause tissue damage due to prolonged UV exposure. In our case, it has been confirmed that up to three repetitions based on the irradiation pattern described in the note of step F2 can be performed without severe damage.
